# Leptin Administration Downregulates the Increased Expression Levels of Genes Related to Oxidative Stress and Inflammation in the Skeletal Muscle of *ob/ob* Mice

**DOI:** 10.1155/2010/784343

**Published:** 2010-06-30

**Authors:** Neira Sáinz, Amaia Rodríguez, Victoria Catalán, Sara Becerril, Beatriz Ramírez, Javier Gómez-Ambrosi, Gema Frühbeck

**Affiliations:** ^1^Metabolic Research Laboratory, Clínica Universidad de Navarra 3, 31008 Pamplona, Spain; ^2^CIBER Fisiopatología de la Obesidad y Nutrición (CIBEROBN), Instituto de Salud Carlos III, Spain; ^3^Department of Endocrinology, Clínica Universidad de Navarra, Pío XII 36, 31008 Pamplona, Spain

## Abstract

Obese leptin-deficient *ob/ob* mice exhibit a low-grade chronic inflammation together with a low muscle mass. Our aim was to analyze the changes in muscle expression levels of genes related to oxidative stress and inflammatory responses in leptin deficiency and to identify the effect of *in vivo* leptin administration. *Ob/ob* mice were divided in three groups as follows: control *ob/ob*, leptin-treated *ob/ob* (1 mg/kg/d) and leptin pair-fed *ob/ob* mice. Gastrocnemius weight was lower in control *ob/ob* than in wild type mice (*P* < .01) exhibiting an increase after leptin treatment compared to control and pair-fed (*P* < .01) *ob/ob* animals. Thiobarbituric acid reactive substances, markers of oxidative stress, were higher in serum (*P* < .01) and gastrocnemius (*P* = .05) of control *ob/ob* than in wild type mice and were significantly decreased (*P* < .01) by leptin treatment. Leptin deficiency altered the expression of 1,546 genes, while leptin treatment modified the regulation of 1,127 genes with 86 of them being involved in oxidative stress, immune defense and inflammatory response. Leptin administration decreased the high expression of *Crybb1, Hspb3, Hspb7, Mt4, Cat, Rbm9, Serpinc1* and *Serpinb1a* observed in control *ob/ob* mice, indicating that it improves inflammation and muscle loss.

## 1. Introduction

Obesity is associated with a low-grade proinflammatory state resulting in an increase of circulating cytokines and inflammatory markers [1]. Inflammatory cytokines have been involved in the impairment of insulin signaling, thus providing molecular links between inflammation and insulin resistance [2]. Inflammation reportedly produces metabolic alterations in skeletal muscle with both inflammatory response and insulin resistance being associated with loss of muscle mass by decreased protein synthesis and increased proteolysis [3–5]. Recently, our group has shown that leptin reverses muscle loss of *ob/ob* mice by inhibiting the activity of the transcriptional factor forkhead box class O3a (FoxO3a) [6]. 

Leptin is an adipocyte-derived peptidic hormone [7] that inhibits food intake and increases thermogenesis by acting through its hypothalamic receptors [8, 9]. Leptin-deficient *ob/ob* mice are obese, hyperphagic, exhibit type 2 diabetes, decreased body temperature and hypogonadotropic hypogonadism [10]. Leptin is a member of the long-chain helical cytokine family and its receptors, which belong to the class I cytokine receptors, are present in bone marrow and spleen as well as on peripheral monocytes and lymphocytes [1]. Leptin increases in response to acute infection and sepsis and it has been reported to exert a profound influence on the function and proliferation of T lymphocytes and natural killer cells [11], on the phagocytosis of macrophages/monocytes [12], and to have a direct effect on the secretion of anti- and proinflammatory cytokines [13]. In this regard, impaired cellular and humoral immunity have been shown in leptin-deficient *ob/ob* mice as well as in leptin receptor-deficient *db/db* mice [14, 15]. These studies reflect the molecular nature of leptin as a cytokine and are consistent with leptin signaling playing a pivotal role in the pathogenesis of obesity-associated inflammation and muscle loss.

In the present paper, gastrocnemius muscle samples from wild type and *ob/ob* mice were analyzed for mRNA presence of over 41,000 transcripts by microarray analysis to identify genes involved in inflammation and oxidative stress that are affected by leptin deficiency and leptin administration in *ob/ob* mice. It was shown that leptin increases the gastrocnemius weight and reduces the high expression levels of genes related to the obesity-associated low-grade inflammation in skeletal muscle of *ob/ob* mice.

## 2. Material and Methods

### 2.1. Animals and Treatments

Ten-week-old male genetically obese *ob/ob* mice (C57BL/6J) (*n* = 15) and their lean control littermates wild type (*n* = 5) supplied by Harlan (Barcelona, Spain) were housed in a room with controlled temperature (22 ± 2°C) and a 12:12 light-dark cycle (lights on at 08:00 am). Body weight of *ob/ob* mice was measured before randomization into control, leptin-treated (1 mg/kg/d) and pair-fed groups (*n* = 5 per group). The control and pair-fed groups received vehicle (PBS), while leptin-treated mice were intraperitoneally administered with leptin (Bachem, Bubendorf, Switzerland) twice daily at 08:00 am and 08:00 pm for 28 days. Control and leptin-treated groups were provided with water and food *ad libitum* with a standard rodent chow (2014S Teklad, Harlan), while daily food intake of the pair-fed group was matched to the amount consumed by the leptin-treated group the day before in order to discriminate the inhibitory effect of leptin on appetite. Animals were sacrificed on the 28th day of treatment by CO_2_ inhalation 20 hours after the last PBS or leptin administration (in order to avoid picking up effects reflecting an acute response) and after 8 hours of fasting. Serum samples and gastrocnemius muscles were obtained and stored at −80°C. All experimental procedures conformed to the European Guidelines for the Care and Use of Laboratory Animals (directive 86/609) and were approved by the Ethical Committee for Animal Experimentation of the University of Navarra (080/05).

### 2.2. Blood Analysis

Serum glucose was analyzed using a sensitive-automatic glucose sensor (Ascensia Elite, Bayer, Barcelona, Spain). Free fatty acid (FFA) concentrations were measured by a colorimetric determination using the NEFA C kit (WAKO Chemicals, Neuss, Germany). Serum glycerol concentrations were evaluated by enzymatic methods as previously described [6]. Serum triglycerides (TG) concentrations were spectrophotometrically determined using a commercial kit (Infinity, Thermo Electron, Melbourne, Australia). Insulin and leptin were determined using specific mouse ELISA kits (Crystal Chem Inc., Chicago, IL, USA). Intra- and interassay coefficients of variation for measurements of insulin and leptin were 3.5% and 6.3%, respectively, for the former, and 2.8% and 5.8%, for the latter. Adiponectin concentrations were also assessed using a mouse ELISA kit (BioVendor Laboratory Medicine, Inc., Modrice, Czech Republic). Intra- and interassay coefficients of variation for adiponectin were 2.6% and 5.3%, respectively. Insulin resistance was calculated using the homeostasis model assessment score (HOMA; fasting insulin (*μ*U/mL) × fasting glucose (mmol/L)/22.5) [16]. An indirect measure of insulin sensitivity was calculated by using the quantitative insulin sensitivity check index (QUICKI; 1/[log(fasting insulin mU/mL) + log(fasting glucose mg/dL)] [17].

Lipid peroxidation was analyzed by the measurement of thiobarbituric acid reactive substances (TBARS) in serum and gastrocnemius as previously described by Conti et al. [18] with some modifications. Since the best-known specific TBARS is malondialdehyde (MDA), we used serum MDA levels, a secondary product of lipid peroxidation, as an indicator of lipid peroxidation and oxidative stress. Gastrocnemius samples (20–30 mg) were homogenized in 20 volumes of phosphate buffer pH 7.4. Serum, muscle homogenates (5 *μ*L) or standard (MDA) were mixed with 120 *μ*L of diethyl thiobarbituric acid (DETBA) 10 mM and vortexed for 5 seconds. The reaction mixture was then incubated at 95°C for 60 minutes. After cooling to room temperature DETBA-MDA adducts were extracted in 360 *μ*L n-butanol vortexing for 1 minute and centrifuged at 1,600 *g* for 10 minutes at room temperature. Then, the chromophore of the DETBA-MDA adduct was quantified in 200 *μ*L of the upper butanol phase by fluorescence emission at 535 nm with an excitation at 590 nm. MDA equivalents (TBARS) were quantified using a calibration curve prepared using MDA standard working solutions and expressed as serum MDA *μ*M and gastrocnemius MDA *μ*M/mg protein. Protein concentrations were determined using a Bradford protein assay kit (BioRad, Hercules, CA, USA).

### 2.3. Microarray Experiments and Analysis

Total RNA was extracted from 20–30 mg of gastrocnemius muscle samples by homogenization with an ULTRA-TURRAX T 25 basic (IKA Werke GmbH, Staufen, Germany) using TRIzol^*™*^ reagent (Invitrogen, Barcelona, Spain). RNA was purified using the RNeasy Mini kit (Qiagen, Barcelona, Spain) and treated with DNase I (RNase-free DNase Set, Qiagen) in order to remove any trace of genomic DNA. 

Gene expression analyses were conducted using the Agilent Whole Mouse Genome array (G4121B, Agilent Technologies, Santa Clara, CA, USA) containing ~41, 000 mouse genes and transcripts. Fluorescence-labeled cDNA probes were prepared from 1 *μ*g of total RNA from each sample (5 animals per group) to be subsequently amino-allyl labeled and amplified using the Amino Allyl MessageAmp II aRNA Amplification Kit (Ambion, Austin, TX, USA). Aliquots (1.2 *μ*g) of amplified aRNA were fluorescently labeled using Cy3/Cy5 (Amersham Biosciences, Buckinghamshire, UK) and then appropriately combined and hybridized to Agilent microarrays. Hybridizations were performed following a reference design, where control samples were pools of RNA from all individual samples. Two hybridizations with fluor reversal (Dye-swap) were performed for each sample. After washing, microarray slides were scanned using a Gene Pix 4100A scanner (Axon Instruments, Union City, CA, USA) and image quantization was performed using the software GenePiX Pro 6.0. Gene expression data for all replicate experiments were analyzed using the GeneSpring GX software version 7.3.1 (Agilent Technologies). Clustering was accomplished with the Gene and Condition Tree algorithms. In addition, Gene Ontology database (http://babelomics.bioinfo.cipf.es) and the KEGG website (http://www.genome.ad.jp/kegg/pathway) were used in conjunction with GeneSpring (http://www.agilent.com/ chem/genespring *) to *identify pathways and functional groups of genes. All microarray data reported are described in accordance with MIAME guidelines (http://www.mged.org/Workgroups/MIAME/miame.html). More information regarding the microarray experiments can be found at the EMBL-European Bioinformatics Institute (http://www.ebi.ac.uk/aerep/login. ArrayExpress accession number: E-MEXP-1831). To validate the microarray data, a number of representative differentially expressed genes were selected to be individually studied by Real-Time PCR (7300 Real Time PCR System, Applied Biosystems, Foster City, CA, USA) (*n* = 5 per group) as previously described [19]. Primers and probes were designed using the software Primer Express 2.0 (Applied Biosystems) and purchased from Genosys (Sigma, Madrid, Spain) (Table 1).

### 2.4. Statistical Analysis

Data are expressed as mean ± standard error of the mean (SEM). Differences between groups were assessed by Kruskal-Wallis followed by Mann Whitney's *U* test. As previously outlined, Gene Ontology groupings were used to identify pathways significantly affected by leptin deficiency as opposed to its administration. Furthermore, statistical comparisons for microarray data to identify differentially expressed genes across different groups were performed using one-way ANOVA and Student's *t*-tests as appropriate. Spearman's correlations were used to evaluate the relations among different variables. All statistical analyses were performed by using the SPSS statistical program version 15.0 for Windows (SPSS, Chicago, IL, USA) and statistical significance was defined as *P* < .05.

## 3. Results

### 3.1. Leptin Treatment Improves the Metabolic Profile of ob/ob Mice

The morphological and biochemical characteristics of wild type and *ob/ob* mice are reported in Table 2. As expected, leptin treatment corrected the obese and diabetic phenotype of *ob/ob* mice. Body weight was significantly higher (*P* < .01) in the control *ob/ob* group as compared to wild type mice. Leptin-treated mice exhibited a decreased body weight (*P* < .01) as compared to control and pair-fed *ob/ob* animals. Importantly, leptin treatment normalized body weight of *ob/ob* mice as compared to wild type (*P* = .690). In addition, the gastrocnemius of control *ob/ob* mice exhibited a lower (*P* < .01) muscle weight than that of wild type mice and it was increased (*P* < .01) by leptin administration in comparison with that of control and pair-fed *ob/ob* rodents. As depicted in Table 2, higher fasting glucose (*P* < .05) and insulin (*P* < .01) concentrations were observed in the control *ob/ob* mice compared to wild types. Although no differences in glucose concentrations were observed in pair-fed as compared to leptin-treated *ob/ob* mice, higher serum insulin concentrations (*P* < .05) were detected in the pair-fed animals than in the leptin-treated *ob/ob* group. Furthermore, leptin administration normalized both the glucose and insulin levels in *ob/ob* mice compared to wild types. These data suggest that leptin increases the insulin sensitivity in peripheral tissues, as evidenced by the lower HOMA and higher QUICKI indices (*P* < .01) in the leptin-treated in comparison with the control *ob/ob* animals. Serum glycerol was markedly increased (*P* < .05) in the control *ob/ob* mice, while FFA and TG levels remained unchanged as compared to wild type mice. Interestingly, leptin not only decreased circulating concentrations of FFA (*P* < .05) and glycerol (*P* < .01) levels as compared to control *ob/ob *mice, but also FFA (*P* < .01), glycerol (*P* < .01) and TG (*P* < .05) concentrations as compared to pair-fed mice. Leptin administration to *ob/ob* mice reduced serum glycerol concentrations (*P* = .032) and tended to decrease FFA (*P* = .095) as compared to wild types. Furthermore, leptin treatment increased the low concentrations of adiponectin of *ob/ob* mice, but the differences fell out of statistical significance (*P* = .095). 

**Figure 1:**
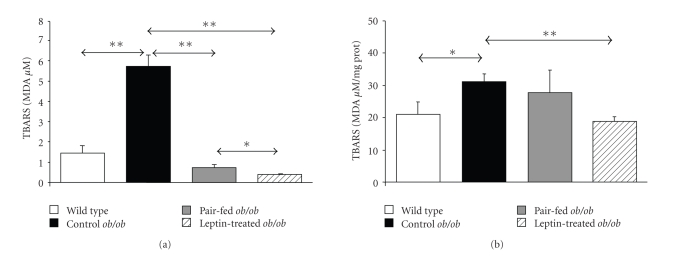
Leptin reduces TBARS concentrations in *ob/ob* mice. Thiobarbituric acid reactive substances (TBARS) presented as concentrations of malondialdehyde (MDA *μ*M) in serum (a) and gastrocnemius muscle (MDA *μ*M/mg prot) (b) of wild type (open), control *ob/ob* (closed), pair-fed *ob/ob* (gray) and leptin-treated *ob/ob* (striped) mice (*n* = 5 per group). Data are expressed as mean ± SEM. *P < .05 and **P < .01 by Kruskal-Wallis followed by Mann Whitney's *U* test.

Control *ob/ob* mice exhibited significantly higher serum TBARS than wild type littermates (*P* < .01), which were significantly reduced after leptin administration as compared to the control (*P* < .01) and pair-fed (*P* < .05) *ob/ob* groups (Figure 1(a)). In addition, leptin decreased (*P* < .01) the high concentrations of MDA measured in the gastrocnemius muscle of control *ob/ob* mice, while this effect was not observed in the pair-fed group (Figure 1(b)). Serum and gastrocnemius TBARS levels were positively associated with body weight, FFA, insulin, and the HOMA index. Oppositely, TBARS levels were negatively associated with adiponectin and the QUICKI index both in serum and muscle. Importantly, a high positive relation were found between serum and gastrocnemius concentrations of TBARS (*ρ* = 0.63, *P* = .003) (Table 3).

### 3.2. Leptin Induces Changes in Gene Expression—Effect of Leptin on Genes Invoved in Oxidative Stress and Inflammation

Differential gene expression profiles in gastrocnemius muscle of wild type and *ob/ob *groups were compared by microarray analysis. Only genes whose mRNA levels were changed 1.5-fold or higher and identified as significantly changed by statistical analysis were designated as differentially expressed genes. Applying these criteria, microarray data showed that 7,582 genes were differentially expressed by leptin deficiency and leptin administration in *ob/ob* mice. In particular, leptin deficiency altered the expression of 1,127 genes between wild type and control *ob/ob* mice. Of these, 580 were upregulated and 547 were downregulated in *ob/ob* mice. Leptin treatment modified the expression of 1,546 genes in *ob/ob* mice, upregulating 512 and repressing 1,034. In addition, leptin repressed 736 genes that were upregulated in gastrocnemius muscle of control *ob/ob* and increased the transcript levels of 846 downregulated genes. Functional enrichment analysis using GeneOntology and KEGG databases revealed that the set of genes with altered expression levels induced by leptin deficiency and administration represents a broad spectrum of biological processes. However, for the purpose of the present paper we focused on the effects of leptin on the set of genes encoding proteins involved in oxidative stress and inflammation. Table 4 shows that leptin deficiency and leptin administration altered the expression of a large number of genes involved in oxidative stress and inflammation. The biological processes mainly affected between control *ob/ob* mice and wild types included “response to oxidative stress” (*P* = .0006), “response to stress” (*P* = .0031) and “acute-phase response” (*P* = .023). Furthermore, several processes regulating proliferation, differentiation, and activity of lymphocytes were also significantly affected by leptin deficiency. Importantly, comparison of leptin-treated and control *ob/ob* groups showed that leptin administration altered the expression of genes implicated in the “positive regulation of lymphocyte activation” (*P* = .0003), “positive regulation of immune response” (*P* = .0032) and “response to stress” (*P* = .0187), as well as genes involved in the “chaperone cofactor dependent protein folding” (*P* = .0023).

**Figure 2:**
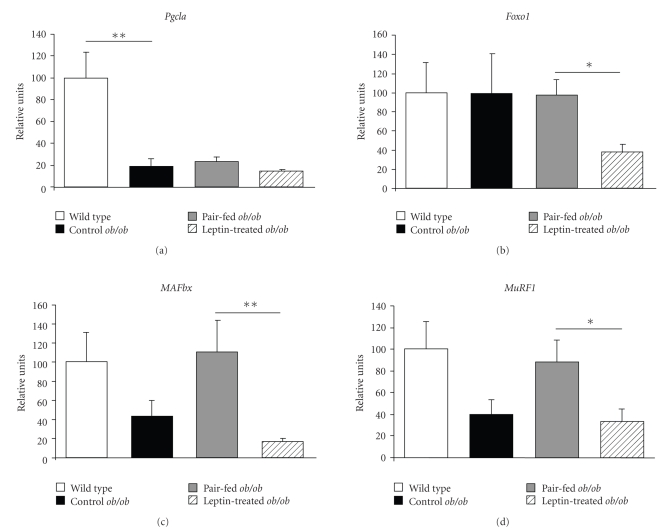
Real-Time PCR analysis of peroxisome proliferator-activated receptor coactivator 1*α* *(Pgc1a)*, forkhead box class O1 *(Foxo1)*, muscle atrophy F box *(MAFbx)* and muscle RING finger 1 *(MuRF1)* in gastrocnemius muscle of wild type (open), control *ob/ob* (closed), pair-fed *ob/ob* (gray) and leptin-treated *ob/ob* (striped) mice (*n* = 5 per group). Data are presented as mean ± SEM of the ratio between gene expression and 18S rRNA. *P < .05 and **P < .01 by Kruskal-Wallis followed by Mann Whitney's *U* test.

Noteworthy, leptin reduced the expression of several genes related to inflammatory conditions. DNA microarray analysis showed that 86 genes encoding proteins related to defense, stress, and inflammatory responses were altered in the gastrocnemius muscle of control *ob/ob* mice and modified by leptin administration. Leptin reduced the mRNA levels of various isoforms of the family of heat shock proteins (HSPs) (*Dnajc16, Dnaja4*, *Dnajb4*, *Hspa2*, *Hspa4*, and *Hspb7*), metallothioneins (*Mt2*, *Mt4*), crystallins (*Cryab*, *Crybb1*) and RNA binding proteins (RBMs) (*Rbm9*, *Rbm22*) in *ob/ob* mice (Table 5). In addition, histocompatibility 2, complement component factor B *H2-Bf* and several genes of the acute-phase response or inflammatory processes, such as kallikrein 5 (*Klk5*), and serine (or cysteine) proteinase inhibitor clade C member 1 (*Serpinc1)* and clade B member 1a (*Serpinb1a*), displayed an increased expression in *ob/ob* mice that was reduced by leptin administration. On the contrary, gene expression of *Cryl1*, *Hsp105*, *Rbm5,* and *H2-Aa* were enhanced in *ob/ob *mice after treated with leptin. Pair-feeding, which accounts for the decrease in food intake that is independent of the direct action of leptin, altered the expression of 1,960 genes, upregulating 984 while downregulating 976 genes. In the context of a food intake reduction as compared to the simple effect due to the caloric restriction, leptin administration further significantly altered the expression of genes involved in processes encompassing “immune response” (*P* = 5.53e^−8^) “defense response” (*P* = 3.83e^−6^), “response to oxidative stress” (*P* = 2.99e^−5^), “positive regulation of T cell activation” (*P* = .0003) and “positive regulation of immune cell mediated cytotoxicity” (*P* = .0004) (Table 4). In particular, the gene array analysis provided evidence for elevated *Hspa4*, *Mt4*, *Crybb1,* and *Serpinb8* mRNA levels in the pair-fed group as compared to the leptin-treated *ob/ob* mice (Table 6). On the contrary, leptin increased the gene expression of *H2-Ab1* and *H2-Eb1* in *ob/ob* mice. To confirm the microarray data, the mRNA expression of several representative transcripts was analyzed by Real-Time PCR (Figure 2). In this sense, leptin administration reduced the mRNA levels of the muscle atrophy-related transcription factor forkhead box O1 (*Foxo1*) and of the E3 ubiquitin-ligases muscle atrophy F-box (*MAFbx*) and muscle RING finger 1 (*MuRF1*) in leptin-treated *ob/ob* mice, while no effect of leptin was evidenced on the mRNA levels of the transcriptional coactivator peroxisome proliferator-activated receptor-*γ* coactivator-1*α* *(* *Pgc1* *α*). The expression of the selected genes was concordant with that of the microarray.

## 4. Discussion

Obesity is accompanied by a chronic proinflammatory state associated not only with insulin resistance, but also with muscular atrophy [4, 5]. Our study provides evidence that leptin constitutes a negative regulator of oxidative stress and inflammation in the gastrocnemius, which is a representative skeletal muscle of the whole skeletal musculature. This statement is supported by findings reported herein: (a) leptin deficiency is accompanied by systemic and skeletal muscle oxidative stress, muscle inflammation, and reduced muscle mass; (b) systemic and skeletal muscle oxidative stress, muscle atrophy and inflammation of *ob/ob* mice are reversed by leptin administration independently of the effects of food intake inhibition. Therefore, leptin is able to prevent the muscle atrophy associated with obese and inflammatory states.

Skeletal muscle constitutes an important target for leptin playing a key role on the regulation of lipid and glucose metabolism [20]. Since obese *ob/ob* mice exhibit an increased oxidative stress and impaired immune response [14, 15] and a reduced skeletal muscle mass [21] compared with their lean littermates, we aimed to identify the genes related to inflammatory processes differentially altered by leptin in the gastrocnemius muscle of obese *ob/ob* mice. In particular, 86 transcripts encoding inflammation-related proteins were shown to be modified by exogenous leptin administration. However, it has to be taken into account that many of these genes are multifunctional and may have important functions in other biological processes. Among them, leptin repressed the high expression levels of acute-phase reactants and several members of the HSP and RBM families. In addition, confirming a previous study of our group [6], leptin treatment increased the reduced muscle weight of gastrocnemius muscle of *ob/ob* mice. Taken together, these data suggest that leptin may prevent the obesity-associated inflammatory state and the muscle mass loss related to inflammatory states in leptin-deficient *ob/ob* mice.

Leptin-deficient *ob/ob* and leptin receptor-deficient *db/db* mice display many abnormalities in the immune response similar to those observed in starved animals and malnourished humans [14, 15, 22]. In this respect, exogenous leptin replacement to *ob/ob* mice modulates T cell responses in mice and prevents starvation-induced immunosuppression, suggesting that lack of leptin is directly involved in these immune system abnormalities [23, 24]. In agreement with these studies, our findings show that leptin deficiency and administration differentially regulate biological processes related to the immune response as well as the T and B cell differentiation and activation in gastrocnemius muscle of *ob/ob* mice.

Oxidative stress is defined as the imbalanced redox state in which prooxidants overwhelm the antioxidant capacity, resulting in an increased production of reactive oxygen species (ROS), ultimately leading to oxidative damage of cellular macromolecules. The major ROS is the superoxide anion (•O_2_^−^). Dismutation of •O_2_^−^ by superoxide dismutase (SOD) produces hydrogen peroxide (H_2_O_2_), a more stable ROS, which, in turn, is converted to water by catalase and glutathione peroxidase (GPx) [25]. Oxidative stress is increased in diabetes [26, 27] with leptin administration reportedly improving insulin sensitivity in normal and diabetic rodents [28–30]. However, the relationship between leptin and oxidative stress has not been clearly exhibited. Leptin stimulates *in vitro* ROS production by inflammatory cells [31] and endothelial cells [32] and the level of systemic oxidative stress in nonobese animals [33, 34], suggesting a “prooxidative” role of leptin. However, administration of recombinant leptin reduces the oxidative stress induced by a high-fat diet in mice [35]. In this sense, findings of our study show a high oxidative stress in diabetic *ob/ob* mice, as reflected by increased TBARS concentrations in serum and the gastrocemius muscle. These observations are in agreement with a large number of studies related to increased plasma TBARS or MDA in diabetic rats [36] and humans [37]. Lipid peroxidation is a common index of free radical mediated injury and induction of antioxidant enzyme is a common cellular response [38]. More importantly, leptin administration decreased serum and gastrocnemius TBARS concentrations as compared to control *ob/ob* mice, with TBARS levels in gastrocnemius muscle from pair-fed *ob/ob* animals remaining very similar to those of control *ob/ob* mice. In this sense, from a molecular perspective, our results further show that transcript levels of *Sod1*, *Gpx3* and glutathione S-transferase *π* 1 *Gstp1 *are downregulated in control *ob/ob* mice as compared to wild type controls being upregulated after leptin treatment. Furthermore, leptin administration also upregulated *Gpx7*, glutathione S-transferase *μ* 5 (*Gstm5*) and glutathione S-transferase *θ* 2 (*Gstt2*). On the contrary, the high expression of catalase (*Cat*) was repressed by the exogenous injection of leptin to *ob/ob* mice. These findings are in line with previous observations showing the restoration of the defective antioxidant enzyme activity in plasma of *ob/ob* mice [39] and humans with a leptin gene mutation [40].

Acute-phase reactants have been suggested to contribute to the maintenance of the chronic low-grade inflammation state involved in the progression of obesity and related diseases [41]. Interestingly, our study provides evidence that genes of the acute-phase response were altered in gastrocnemius muscle of *ob/ob* mice, which were counteracted by exogenous leptin administration. Leptin reduced the elevated gene expression of tissue-type plasminogen activator (*Plat*) and lipocalin-2 (*Lcn2*), which are upregulated in many inflammatory conditions [42, 43], including human obesity [44]. In addition, a pivotal role for oxidative stress in the pathogenesis of muscle wasting in disuse and in a variety of pathological conditions is now being widely recognized [45]. A potential link between oxidative stress and muscle atrophy involves the redox regulation of the proteolytic system [46]. Moreover, various inflammatory cytokines induce oxidative stress [47] and muscle atrophy through the activation of the lysosomal [48, 49] and the ubiquitin-proteolysis system [50]. In this context, biological processes related to oxidative stress and inflammatory responses were altered in the gastrocnemius muscle of *ob/ob* mice and improved following leptin treatment. In spite of the usual upregulation of the E3 ubiquitin-ligases MAFbx and MuRF1 in most conditions associated with atrophy, their gene expression levels in *ob/ob* were lower as compared to wild type animals, although no statistically significant differences were observed. Contrarily to what would be expected, leptin administration prevented the increase of both MAFbx and MuRF1 mRNA expression levels induced by pair-feeding in *ob/ob* mice. A plausible explanation for this surprising finding may relate to the fact that in extreme conditions the energy homeostasis system is overriden whereby leptin is able to inhibit muscular protein degradation associated to food intake reduction. These data are in accordance with a previous study of our group evidencing that leptin replacement inhibits the ubiquitin proteolysis system activity in leptin-deficient mice [6]. Muscle atrophy is associated with increased expression of genes coding for RBM proteins which facilitate the translation, protection, and restoration of native RNA conformations during oxidative stress. It has been suggested that the gene expression of RBM proteins may increase as a compensatory mechanism in response to loss of muscle proteins [51, 52]. Other proteins involved in oxidative stress are metallothioneins, endogenous antioxidants [53] that have been shown to be overexpressed in muscle atrophy in rodents [54–56]. In the present work, we have observed that administration of leptin inhibits the gene expression of several members of the RBM (*Rbm9*, *Rbm22*) and metallothioneins (*Mt2*, *Mt4*) families in the gastrocnemius of *ob/ob* mice, suggesting that leptin may modulate the inflammatory and oxidative stress responses and consequently, the muscle loss related to inflammatory states.

Genes involved in the chaperone system were also differentially expressed in *ob/ob* mice as compared to wild types and modified by leptin treatment. HSPs represent a family of molecular chaperones induced in response to cellular stress, responsible for maintaining the structure of proteins and contributing to the repair of damaged or malformed proteins in highly oxidative and lipotoxic conditions. As a result, HSPs are considered antiproteolytic proteins [57]. Muscle atrophy is also associated with an increased gene expression of HSPs [58]. In fact, HSPs are repressed in many rat models of skeletal muscle atrophy [54, 59, 60]. HSP70 is constitutively expressed in skeletal muscle, but its levels are increased in response to oxidative stress [61] with the induction of HSP70 expression by hyperthermia and during inactivity attenuating muscle atrophy [62, 63]. In this regard, a recent study has shown that HSP70 prevents muscle atrophy induced by physical inactivity through inhibition of the muscle atrophy-related transcription factor FoxO3a and the expression of MAFbx and MuRF1 [64]. Among the HSPs, HSP70 and *α*B-crystallin in particular, are considered negative regulators of muscle cell apoptosis [65, 66] and may inhibit the loss of nuclei taking place during muscle atrophy. In addition, ROS induce the activity of FoxO [67] and gene expression of members of the ubiquitin-proteolysis system in myotubes [68]. In this sense, our results provide evidence that leptin inhibits the increased gene expression of different members of the HSPs (*Hspb7*, *Dnajc16*, *Hspa4*, *Cryab,* and *Crybb1*) in the gastrocnemius muscle of *ob/ob* mice. Taken together, the elevated expression of HSPs in the control and pair-fed *ob/ob* groups suggests a high defense and stress response in these mice. Moreover, induction of HSPs may confer broader health benefits to patients who are insulin resistant or diabetic [69]. In mammals, caloric restriction has been shown to upregulate HSP induction [70, 71], while expression of HSP72 has been found to be low in skeletal muscle of patients with insulin resistance or type 2 diabetes [72, 73]. Figueiredo et al. [74] have recently shown that leptin downregulates HSP70 gene expression in chicken liver and hypothalamus but not in muscle, which was independent of food intake restriction. On the contrary, Bonior et al. [75] reported an increase in HSP60 gene expression in pancreatic cells by leptin. 

Obesity is accompanied by a chronic proinflammatory state resulting in an increase in circulating cytokines and inflammatory markers. In this regard, inflammation produces metabolic alterations in skeletal muscle with both inflammatory response and insulin resistance being associated with muscle mass loss. Findings of our study provide evidence that systemic and skeletal muscle oxidative stress, muscle atrophy and the elevated expression of genes involved in oxidative stress and inflammation of *ob/ob* mice are reversed by leptin administration. Taken together, these data thereby support that leptin is able to prevent the muscle atrophy associated with obese and inflammatory states in *ob/ob* mice. Most obese people develop muscle atrophy in spite of exhibiting high leptin circulating concentrations, which may be explained by the leptin resistance present in these patients. Our paper sheds light on the relation between obesity and the loss of muscle mass associated to inflammatory states suggesting that leptin treatment may be an attractive therapeutic approach to prevent muscle loss associated with inflammatory diseases.

## Figures and Tables

**Table 1:** Sequences of the primers and Taqman probes used in the Real-Time PCR.

Gene	Gene Symbol	GenBank accesión number	Oligonucleotide sequence (5′-3′)
Peroxisome proliferator-activated receptor-*γ* coactivator-1*α*	*Pgc1a*	NM_008904	Forward: GTCTGAAAGGGCCAAACAGAGA
			Reverse: TCAATTCTGTCCGCGTTGTG
			Probe: FAM-AGCAGAAAGCAATTGAAGAGCGCCGT-TAMRA
Forkhead box O1	*Foxo1*	NM_019739	Forward: GCGGGCTGGAAGAATTCAAT
			Reverse: TCCTTCATTCTGCACTCGAATAAAC T
			Probe: FAM-CGCCACAATCTGTCCCTTCACA-TAMRA
Muscle atrophy F box	*MAFbx*	NM_026346	Forward: CCATCCTGGATTCCAGAAGATTC
			Reverse: TCAGGGATGTGAGCTGTGACTTT
			Probe: FAM-CTACGTAGTAAGGCTGTTGGAGCTGAT-TAMRA
Muscle RING finger 1	*MuRF1*	NM_001039048	Forward: CGCCATGAAGTGATCATGGA
			Reverse: TCCTTGGAAGATGCTTTGCA
			Probe: FAM-TGTACGGCCTGCAGAGGAACCTGAAA-TAMRA

**Table 2:** Total body and skeletal muscle weights and biochemical characteristics of wild type and *ob/ob* mice.

	wild type	control *ob/ob *	pair-fed *ob/ob *	leptin-treated *ob/ob *
Body weight (g)	25.6 ± 0.3	47.8 ± 4.9^b^	35.7 ± 0.7	24.7 ± 1.2^d,f^
Gastrocnemius (mg)	142.9 ± 3.4	90.7 ± 10.0^b^	68.5 ± 1.6	104.9 ± 2.6^b,f^
Gastrocnemius (mg/g)	5.59 ± 0.12	1.91 ± 0.11^b^	1.92 ± 0.07	4.28 ± 0.15^b,d,f^
Glucose (mg/dL)	149 ± 42	430 ± 59^a^	160 ± 24^d^	178 ± 29^d^
FFA (mmol/L)	1.62 ± 0.49	1.61 ± 0.30	1.65 ± 0.12	0.78 ± 0.13^c,f^
Glycerol (mmol/L)	42.8 ± 6.7	81.6 ± 19.6^a^	39.6 ± 4.9^c^	12.3 ± 4.7^a,d,f^
TG (mg/dL)	122 ± 18	169 ± 32	151 ± 10	86 ± 17^e^
Insulin (ng/mL)	0.42 ± 0.09	8.60 ± 1.51^b^	2.40 ± 0.68^c^	0.47 ± 0.09^d,e^
Adiponectin (*μ*g/mL)	30.2 ± 3.0	28.3 ± 5.4	39.1 ± 1.8	40.2 ± 3.0
Leptin (ng/mL)	1.36 ± 0.42	UD	UD	3.48 ± 1.02
HOMA	4.3 ± 1.8	202.4 ± 33.8^b^	25.8 ± 10.4^d^	5.12 ± 1.1^d^
QUICKI	0.333 ± 0.023	0.205 ± 0.003^b^	0.263 ± 0.015^d^	0.311 ± 0.016^d^

Data are mean ± SEM (*n* = 5 per group). Differences between groups were analyzed by Kruskal-Wallis followed by Mann Whitney's *U* test. ^a^ *P* < .05 and ^b^ *P* < .01*versus* wild type. ^c^ *P* < .05 and ^d^ *P* < .01 *versus ob/ob*. ^e^ *P* < .05 and ^f^ *P* < .01* versus* pair-fed *ob/ob*. FFA: free fatty acids. TG: triglycerides. UD: undetectable. HOMA: homeostasis model assessment. QUICKI: quantitative insulin sensitivity check index.

**Table 3:** Bivariate analysis of the correlations between TBARS concentrations in serum and the gastrocnemius muscle with anthropometric and biochemical variables in wild type and *ob/ob* mice.

	Serum	TBARS	Gastrocnemius	TBARS
	*ρ*	*P*	*ρ*	*P*
Body weight	0.57	.009	0.46	.040
Glucose	0.44	.055	0.38	.103
FFA	0.54	.015	0.59	.007
Glycerol	0.49	<.001	0.44	.053
TG	0.44	.054	0.44	.050
Insulin	0.49	.027	0.52	.020
Adiponectin	−0.51	.022	−0.53	.016
QUICKI	−0.48	.031	−0.48	.033
HOMA	0.53	.019	0.51	.025

Values are Spearman's correlation coefficients (*ρ*) and associated *P* values. TBARS: thiobarbituric acid reactive substances. FFA: free fatty acids. TG: triglycerides. HOMA: homeostasis model assessment. QUICKI: quantitative insulin sensitivity check index.

**Table 4:** Biological processes according to Gene Ontology (GO) and number of genes altered by leptin deficiency, leptin administration, and pair-feeding in the gastrocnemius muscle of wild type and *ob/ob* mice.

Category	Genes in Category	wild type *vs ob/ob *		*ob/ob vs* leptin		leptin* vs* pair-fed	
		Altered genes	*P* value	Altered genes	*P* value	Altered genes	*P* value
GO:6950: response to stress	**1156**	**61**	**.00314**	**69**	**.0187**	**22**	.0757
GO:6952: defense response	**1010**	**43**	.182	**47**	.510	**33**	**3.83** **e** ^−6^
GO:6955: immune response	**835**	**36**	.186	**45**	.165	**33**	**5.53** **e** ^−8^
GO:45321: immune cell activation	**230**	**9**	.475	**13**	.270	**6**	.0974
GO:46649: lymphocyte activation	**208**	**9**	.359	**13**	.170	**6**	.0673
GO:6954: inflammatory response	**199**	**4**	.938	**4**	.984	**2**	.7590
GO:50776: regulation of immune response	**148**	**9**	.097	**12**	**.0426**	**8**	**.00102**
GO:6959: humoral immune response	**123**	**7**	.169	**8**	.211	**4**	.0891
GO:42110: T cell activation	**112**	**5**	.396	**7**	.263	**5**	**.0191**
GO:30098: lymphocyte differentiation	**107**	**8**	**.0441**	**8**	.123	**4**	.0597
GO:42113: B cell activation	**101**	**3**	.724	**7**	.188	**3**	.1610
GO:6800: oxygen and reactive oxygen species metabolism	**92**	**11**	**.00056**	**7**	.135	**7**	**.00027**
GO:50778: positive regulation of immune response	**91**	**7**	.0508	**11**	**.0032**	**8**	**3.6** **e** ^−5^
GO:51249: regulation of lymphocyte activation	**89**	**7**	**.046**	**10**	**.00808**	**5**	**.0076**
GO:19882: antigen presentation	**81**	**9**	**.0029**	**9**	**.0125**	**8**	**1.53** **e** ^−5^
GO:31098: stress-activated protein kinase signaling pathway	**80**	**8**	**.00921**	**5**	.313	**1**	.6690
GO:30333: antigen processing	**78**	**11**	**.00013**	**13**	**5.65** **e** ^−5^	**8**	**1.16** **e** ^−5^
GO:7254: JNK cascade	**75**	**8**	**.00629**	**4**	.461	**1**	.6450
GO:46651: lymphocyte proliferation	**67**	**2**	.712	**5**	.199	**2**	.2340
GO:6979: response to oxidative stress	**65**	**9**	**.0006**	**7**	**.0303**	**7**	**2.99** **e** ^−5^
GO:50863: regulation of T cell activation	**62**	**5**	.0779	**6**	.0667	**5**	**.0016**
GO:7249: I-kappaB kinase/NF-kappaB cascade	**61**	**2**	.663	**3**	.542	**3**	.0512
GO:51251: positive regulation of lymphocyte activation	**58**	**6**	**.0196**	**10**	**.0003**	**5**	**.00118**
GO:30217: T cell differentiation	**54**	**5**	**.0481**	**6**	**.0380**	**4**	**.00638**
GO:9266: response to temperature stimulus	**54**	**12**	**4.78** **e** ^−7^	**13**	**7.96** **e** ^−7^	**1**	.5260
GO:30183: B cell differentiation	**50**	**2**	.554	**3**	.410	**2**	.1500
GO:50670: regulation of lymphocyte proliferation	**46**	**2**	.509	**3**	.360	**1**	.4700
GO:50864: regulation of B cell activation	**46**	**2**	.509	**5**	.0606	**2**	.1310
GO:42087: cell-mediated immune response	**44**	**1**	.809	**1**	.876	**2**	.1220
GO:50777: negative regulation of immune response	**43**	**3**	.210	**2**	.599	**1**	.4480
GO:50870: positive regulation of T cell activation	**43**	**5**	**.0203**	**6**	**.0137**	**5**	**.000294**
GO:42088: T-helper 1 type immune response	**41**	**1**	.786	**1**	.857	**2**	.1080
GO:9408: response to heat	**40**	**9**	**1.17** **e** ^−5^	**12**	**1.54** **e** ^−7^	**1**	.4240
GO:45619: regulation of lymphocyte differentiation	**36**	**6**	**.00186**	**5**	**.0242**	**4**	**.00144**
GO:42100: B cell proliferation	**32**	**1**	.699	**5**	**.0150**	**2**	.0709
GO:19884: antigen presentation, exogenous antigen	**31**	**9**	**1.17** **e** ^−6^	**9**	**7.62** **e** ^−6^	**8**	**6.81** **e** ^−9^
GO:50851: antigen receptor-mediated signaling pathway	**30**	**1**	.676	**3**	.160	**1**	.3390
GO:50871: positive regulation of B cell activation	**30**	**1**	.676	**5**	**.0115**	**2**	.0633
GO:51250: negative regulation of lymphocyte activation	**30**	**2**	.304	**1**	.759	**1**	.3390
GO:50671: positive regulation of lymphocyte proliferation	**29**	**2**	.290	**3**	.149	**1**	.3300
GO:1909: immune cell mediated cytotoxicity	**27**	**2**	.262	**2**	.358	**3**	**.00584**
GO:45580: regulation of T cell differentiation	**26**	**5**	**.00232**	**5**	**.00617**	**4**	**.00041**
GO:30888: regulation of B cell proliferation	**24**	**1**	.594	**3**	.0975	**1**	.2820
GO:45621: positive regulation of lymphocyte differentiation	**22**	**4**	**.00788**	**5**	**.00288**	**3**	**.00323**
GO:19886: antigen processing, exogenous antigen via MHC class II	**21**	**9**	**2.37** **e** ^−8^	**8**	**2.45** **e** ^−6^	**8**	**1.98** **e** ^−10^
GO:45058: T cell selection	**20**	**2**	.167	**1**	.613	**3**	**.00244**
GO:50868: negative regulation of T cell activation	**20**	**1**	.528	**1**	.613	**1**	.2410
G O:42591: antigen presentation, exogenous antigen via MHC class II	**19**	**6**	**4.42** **e** ^−5^	**6**	**.000157**	**6**	**1.47** **e** ^−7^
GO:45582: positive regulation of T cell differentiation	**19**	**4**	**.00456**	**5**	**.00143**	**3**	**.0021**
GO:1910: regulation of immune cell mediated cytotoxicity	**18**	**2**	.141	**2**	.202	**3**	**.00178**
GO:19724: B cell mediated immunity	**18**	**1**	.491	**1**	.574	**1**	.2200
GO:45577: regulation of B cell differentiation	**16**	**1**	.452	**1**	.532	**2**	**.0198**
GO:46328: regulation of JNK cascade	**16**	**1**	.452	**2**	.168	**1**	.1980
GO:30890: positive regulation of B cell proliferation	**14**	**1**	.409	**3**	**.0246**	**1**	.1760
GO:45060: negative thymic T cell selection	**14**	**1**	.409	**1**	.485	**1**	.1760
GO:51085: chaperone cofactor dependent protein folding	**13**	**2**	.0809	**4**	**.00234**	**3**	**.00066**
GO:1912: positive regulation of immune cell mediated cytotoxicity	**11**	**1**	.338	**1**	.407	**3**	**.00039**
GO:48002: antigen presentation, peptide antigen	**10**	**5**	**1.45** **e** ^−5^	**5**	**4.39** **e** ^−5^	**4**	**6.8** **e** ^−6^
GO:48005: antigen presentation, exogenous peptide antigen	**7**	**5**	**1.33** **e** ^−6^	**5**	**4.11** **e** ^−6^	**4**	**1.17** **e** ^−6^
GO:45620: negative regulation of lymphocyte differentiation	**6**	**2**	**.0184**	**1**	.248	**1**	.0794
GO:46330: positive regulation of JNK cascade	**4**	**1**	.139	**1**	.173	**1**	.0537
GO:45581: negative regulation of T cell differentiation	**2**	**1**	.0723	**1**	.0905	**1**	**.0272**

*P* values reflect the significance of change in prevalence of genes in each category under the leptin deficiency (*ob/ob*), leptin administration (leptin) and pair-feeding (pair-fed) conditions in *ob/ob* mice to the expected prevalence of genes in each category. Statistically significant *P* values are highlighted in bold.

**Table 5:** Genes involved in oxidative stress and inflammatory responses altered by leptin in the gastrocnemius muscle of *ob/ob* mice.

GeneBank Number	Gene Symbol	Gene Name	Fold change		Ratio
			*ob/ob*	leptin	
Genes downregulated by leptin					

NM_009804	*Cat*	*Catalase*	1.47	1.13	0.77
NM_007705	*Cirbp*	*Cold inducible RNA binding protein*	1.68	1.14	0.68
NM_009964	*Cryab*	*Crystallin, * *α* * B*	1.32	1.15	0.87
NM_023695	*Crybb1*	*Crystallin, * *β* * B1*	2.21	1.39	0.63
NM_023646	*Dnaja3*	*DnaJ (Hsp40) homolog, subfamily A, member 3*	0.95	0.64	0.67
NM_021422	*Dnaja4*	*Heat shock protein, DNAJ-like 4*	0.88	0.30	0.34
NM_018808	*Dnajb1*	*DnaJ (Hsp40) homolog, subfamily B, member 1*	0.44	0.33	0.74
NM_026400	*Dnajb11*	*DnaJ (Hsp40) homolog, subfamily B, member 11*	1.11	0.93	0.84
NM_027287	*Dnajb4*	*DnaJ (Hsp40) homolog, subfamily B, member 4*	1.09	0.60	0.55
NM_019874	*Dnajb5*	*DnaJ (Hsp40) homolog, subfamily B, member 5*	1.03	0.73	0.72
NM_011847	*Dnajb6*	*DnaJ (Hsp40) homolog, subfamily B, member 6 isoform c*	0.70	0.47	0.67
NM_013760	*Dnajb9*	*DnaJ (Hsp40) homolog, subfamily B, member 9*	0.62	0.39	0.63
NM_007869	*Dnajc1*	*DnaJ (Hsp40) homolog, subfamily C, member 1*	0.82	0.52	0.63
NM_028873	*Dnajc14*	*DnaJ (Hsp40) homolog, subfamily C, member 14*	1.12	0.87	0.77
NM_172338	*Dnajc16*	*DnaJ (Hsp40) homolog, subfamily C, member 16*	1.15	0.66	0.57
NM_009584	*Dnajc2*	*DnaJ (Hsp40) homolog, subfamily C, member 2*	1.01	0.82	0.81
NM_008929	*Dnajc3*	*DnaJ (Hsp40) homolog, subfamily C, member 3B*	1.02	0.83	0.82
NM_016775	*Dnajc5*	*DnaJ (Hsp40) homolog, subfamily C, member 5*	0.74	0.50	0.67
NM_010344	*Gsr*	*Glutathione reductase 1*	1.17	0.71	0.61
NM_008180	*Gss*	*Glutathione synthetase*	1.13	0.88	0.78
NM_010357	*Gsta4*	*Glutathione S-transferase, * *α* * 4*	1.50	1.46	0.97
NM_010362	*Gsto1*	*Glutathione S-transferase *ο* 1*	1.42	1.15	0.81
NM_008198	*H2-Bf*	*Histocompatibility 2, complement component factor B*	2.00	1.44	0.72
NM_013558	*Hspa1l*	*Heat shock 70kDa protein 1-like*	1.60	1.04	0.65
NM_008301	*Hspa2*	*Heat shock protein 2*	1.49	0.98	0.65
NM_008300	*Hspa4*	*Heat shock protein 4*	0.92	0.30	0.32
NM_031165	*Hspa8*	*Heat shock protein 8*	0.91	0.57	0.62
NM_010481	*Hspa9a*	*Heat shock protein 9*	1.03	0.88	0.86
NM_024441	*Hspb2*	*Heat shock protein 2*	1.45	1.21	0.83
NM_019960	*Hspb3*	*Heat shock protein 3*	1.66	1.27	0.77
NM_013868	*Hspb7*	*Heat shock protein family, member 7*	1.83	0.35	0.19
NM_008302	*Hspcb*	*Heat shock protein 1, * *β*	0.86	0.69	0.80
NM_008416	*Junb*	*Jun-B oncogene*	0.59	0.36	0.61
NM_010592	*Jund1*	*Jun D proto-oncogene*	1.49	0.94	0.63
NM_008456	*Klk5*	*Kallikrein 5*	2.23	1.43	0.64
NM_026346	*MAFbx*	*Muscle atrophy F box*	0.65	0.43	0.67
NM_008209	*Mr1*	*Histocompatibility-2 complex class 1-like*	1.19	0.98	0.82
NM_008630	*Mt2*	*Metallothionein 2*	1.11	0.50	0.46
NM_008631	*Mt4*	*Metallothionein 4*	1.27	1.03	0.81
NM_008872	*Plat*	*Plasminogen activator, tissue*	1.56	1.12	0.72
NM_029397	*Rbm12*	*RNA binding motif protein 12*	1.40	1.03	0.74
NM_026453	*Rbm13*	*RNA binding motif protein 13*	1.01	0.87	0.86
NM_026434	*Rbm18*	*RNA binding motif protein 18*	0.94	0.59	0.63
BC080205	*Rbm22*	*RNA binding motif protein 22*	1.14	0.75	0.66
BC040811	*Rbm28*	*Rbm28 protein*	0.69	0.49	0.71
NM_172762	*Rbm34*	*RNA binding motif protein 34*	1.01	0.67	0.66
NM_009032	*Rbm4*	*RNA binding motif protein 4*	1.04	0.81	0.78
NM_148930	*Rbm5*	*RNA binding motif protein 5*	0.69	0.63	0.91
NM_144948	*Rbm7*	*RNA binding motif protein 7*	0.81	0.74	0.91
NM_025875	*Rbm8a*	*RNA binding motif protein 8a*	0.91	0.69	0.76
NM_175387	*Rbm9*	*RNA binding motif protein 9 isoform 2*	1.96	0.46	0.23
NM_025429	*Serpinb1a*	*Serine (or cysteine) proteinase inhibitor, clade B, member 1a*	2.73	2.09	0.77
NM_080844	*Serpinc1*	*Serine (or cysteine) proteinase inhibitor, clade C (antithrombin), member 1*	4.98	1.93	0.39
NM_008871	*Serpine1*	*Serine (or cysteine) proteinase inhibitor, clade E, member 1*	2.12	0.97	0.46
NM_011340	*Serpinf1*	*Serine (or cysteine) proteinase inhibitor, clade F, member 1*	2.43	1.50	0.62
NM_009776	*Serping1*	*Serine (or cysteine) proteinase inhibitor, clade G, member 1*	1.41	1.15	0.81
NM_009776	*Serping1*	*Serine (or cysteine) proteinase inhibitor, clade G, member 1*	1.41	1.15	0.81
NM_013749	*Tnfrsf12a*	*Tumor necrosis factor receptor superfamily, member 12a*	0.78	0.29	0.37

Genes upregulated by leptin					

NM_030004	*Cryl1*	*Crystallin * *λ* * 1*	1.25	1.72	1.38
NM_016669	*Crym*	*Crystallin * *μ*	1.37	1.64	1.19
NM_133679	*Cryzl1*	*Crystallin, * *ζ* * (quinone reductase)-like 1*	1.10	1.28	1.16
NM_008161	*Gpx3*	*Glutathione peroxidase 3 isoform 2*	0.47	0.54	1.15
NM_024198	*Gpx7*	*Glutathione peroxidase 7*	1.00	1.34	1.33
NM_010359	*Gstm3*	*Glutathione S-transferase, * *μ* * 3*	1.06	1.23	1.17
NM_010360	*Gstm5*	*Glutathione S-transferase, * *μ* * 5*	1.09	1.39	1.27
NM_013541	*Gstp1*	*Glutathione S-transferase, * *π* * 1*	0.87	1.04	1.20
NM_010361	*Gstt2*	*Glutathione S-transferase, * *θ* * 2*	1.21	1.70	1.40
NM_133994	*Gstt3*	*Glutathione S-transferase, * *θ* * 3*	1.53	1.69	1.11
NM_010363	*Gstz1*	*Glutathione transferase zeta 1 (maleylacetoacetate isomerase)*	1.13	1.24	1.10
NM_010378	*H2-Aa*	*Histocompatibility 2, class II antigen A, * *α*	0.46	1.26	2.76
NM_010379	*H2-Ab1*	*Histocompatibility 2, class II antigen A, * *β* *1*	0.37	1.04	2.84
NM_010382	*H2-Eb1*	*Histocompatibility 2, class II antigen E * *β*	0.43	1.03	2.40
NM_010395	*H2-T10*	*Histocompatibility 2, T region locus 10*	1.11	1.41	1.27
NM_013559	*Hsp105*	*Heat shock protein 105*	0.41	0.73	1.79
NM_008303	*Hspe1*	*Heat shock protein 1 (chaperonin 10)*	0.67	0.98	1.48
AK_052911	*MuRF1*	*M muscle RING finger 1*	0.20	0.28	1.43
XM_131139	*Rbm15*	*RNA binding motif protein 15*	0.81	1.34	1.66
NM_197993	*Rbm21*	*RNA binding motif protein 21*	0.67	0.73	1.08
BC029079	*Rbm26*	*Rbm26 protein*	0.75	1.19	1.59
AK087759	*Rbm27*	*RNA binding motif protein 27*	0.88	1.19	1.36
NM_148930	*Rbm5*	*RNA binding motif protein 5*	0.77	1.18	1.55
NM_011251	*Rbm6*	*RNA binding motif protein 6 isoform a*	0.80	0.97	1.21
NM_207105	*Rmcs1*	*histocompatibility 2, class II antigen A, * *β* *1*	0.38	0.89	2.37
NM_011454	*Serpinb6b*	*Serine (or cysteine) proteinase inhibitor, clade B, member 6b*	1.06	1.23	1.16
NM_009825	*Serpinh1*	*Serine (or cysteine) proteinase inhibitor, clade H, member 1*	0.65	0.99	1.53
NM_145533	*Smox*	*Spermine oxidase*	0.41	1.23	3.00
AK080908	*Sod1*	*Superoxide dismutase*	0.58	0.62	1.07
NM_011723	*Xdh*	*Xanthine dehydrogenase*	0.68	1.01	1.47

Differential expression of genes is indicated as fold changes with respect to the wild type group presenting only the genes which were significantly different (*P* < .05) between the leptin-treated and the *ob/ob* groups. Ratio: fold change value for leptin-treated between the *ob/ob* groups.

**Table 6:** Genes involved in oxidative stress and inflammatory responses altered by leptin in gastrocnemius muscle of *ob/ob *mice independently of food intake restriction.

GeneBank Number	Gene symbol	Gene name	*Fold change*
Genes downregulated by leptin			

NM_023695	*Crybb1*	*Crystallin, * *β* * B1*	0.51
NM_021422	*Dnaja4*	*Heat shock protein, DNAJ-like 4*	0.63
NM_019739	*Foxo1*	*Forkhead box O1*	0.34
NM_008300	*Hspa4*	*Heat shock protein 4*	0.64
NM_013868	*Hspb7*	*Heat shock protein family, member 7*	0.34
NM_010592	*Jund1*	*Jun D proto-oncogene*	0.50
NM_008456	*Klk5*	*Kallikrein 5*	0.46
NM_008491	*Lcn2*	*Lipocalin 2*	0.34
NM_008631	*Mt4*	*Metallothionein 4*	0.63
NM_026346	*MAFbx*	*Muscle atrophy F box*	0.37
AK_052911	*MuRF1*	*M muscle RING finger 1*	0.29
NM_011459	*Serpinb8*	*Serine (or cysteine) proteinase inhibitor, clade B, member 8*	0.38
NM_011459	*Serpinb8*	*Serine (or cysteine) proteinase inhibitor, clade B, member 8*	0.59
NM_008871	*Serpine1*	*Serine (or cysteine) proteinase inhibitor, clade E, member 1*	0.42

Genes upregulated by leptin			

NM_009735	*B2m*	*β* *-2-microglobulin*	1.92
NM_010361	*Gstt2*	*Glutathione S-transferase, * *θ* * 2*	1.94
NM_010379	*H2-Ab1*	*Histocompatibility 2, class II antigen A, * *β* * 1*	4.72
NM_010379	*H2-Ab1*	*Histocompatibility 2, class II antigen A, * *β* * 1*	3.66
NM_010386	*H2-DMa*	*Histocompatibility 2, class II, locus Dma*	2.35
NM_010387	*H2-DMb1*	*Histocompatibility 2, class II, locus Mb1*	3.31
NM_010382	*H2-Eb1*	*Histocompatibility 2, class II antigen E * *β*	4.65
NM_013559	*Hsp105*	*Heat shock protein 105*	1,79
AK220167	*Hspa4*	*MKIAA4025 protein*	1,59
NM_207105	*Rmcs1*	*Histocompatibility 2, class II antigen A, * *β* * 1*	4.24
NM_207105	*Rmcs1*	*Histocompatibility 2, class II antigen A, * *β* * 1*	4.17
NM_009255	*Serpine2*	*Serine (or cysteine) proteinase inhibitor, clade E, member 2*	1.53
NM_009825	*Serpinh1*	*Serine (or cysteine) proteinase inhibitor, clade H, member 1*	2.21
NM_145533	*Smox*	*Spermine oxidase*	4.67

Differential expression of genes is indicated as fold changes presenting only the genes which were significantly different (*P* < .05) between the leptin-treated and the pair-fed *ob/ob* groups.

## References

[1] Fantuzzi G, Faggioni R (2000). Leptin in the regulation of immunity, inflammation, and hematopoiesis. *Journal of Leukocyte Biology*.

[2] Andersson CX, Gustafson B, Hammarstedt A, Hedjazifar S, Smith U (2008). Inflamed adipose tissue, insulin resistance and vascular injury. *Diabetes/Metabolism Research and Reviews*.

[3] Wang X, Hu Z, Hu J, Du J, Mitch WE (2006). Insulin resistance accelerates muscle protein degradation: activation of the ubiquitin-proteasome pathway by defects in muscle cell signaling. *Endocrinology*.

[4] Schaap LA, Pluijm SMF, Deeg DJH, Visser M (2006). Inflammatory markers and loss of muscle mass (Sarcopenia) and strength. *American Journal of Medicine*.

[5] Powers SK, Kavazis AN, McClung JM (2007). Oxidative stress and disuse muscle atrophy. *Journal of Applied Physiology*.

[6] Sáinz N, Rodríguez A, Catalán V (2009). Leptin administration favors muscle mass accretion by decreasing FoxO3a and increasing PGC-1*α* in *ob/ob* mice. *PLoS ONE*.

[7] Zhang Y, Proenca R, Maffei M, Barone M, Leopold L, Friedman JM (1994). Positional cloning of the mouse obese gene and its human homologue. *Nature*.

[8] Friedman JM, Halaas JL (1998). Leptin and the regulation of body weight in mammals. *Nature*.

[9] Frühbeck G, Gómez-Ambrosi J (2001). Rationale for the existence of additional adipostatic hormones. *FASEB Journal*.

[10] Pelleymounter MA, Cullen MJ, Baker MB (1995). Effects of the obese gene product on body weight regulation in *ob/ob* mice. *Science*.

[11] Otero M, Lago R, Lago F (2005). Leptin, from fat to inflammation: old questions and new insights. *FEBS Letters*.

[12] Mancuso P, Gottschalk A, Phare SM, Peters-Golden M, Lukacs NW, Huffnagle GB (2002). Leptin-deficient mice exhibit impaired host defense in Gram-negative pneumonia. *Journal of Immunology*.

[13] Loffreda S, Yang SQ, Lin HZ (1998). Leptin regulates proinflammatory immune responses. *FASEB Journal*.

[14] Mandel MA, Mahmoud AAF (1978). Impairment of cell-mediated immunity in mutation diabetic mice (*db/db*). *Journal of Immunology*.

[15] Chandra RK (1980). Cell-mediated immunity in genetically obese (C57BL/6J *ob/ob*) mice. *American Journal of Clinical Nutrition*.

[16] Matthews DR, Hosker JP, Rudenski AS (1985). Homeostasis model assessment: insulin resistance and *β*-cell function from fasting plasma glucose and insulin concentrations in man. *Diabetologia*.

[17] Katz A, Nambi SS, Mather K (2000). Quantitative insulin sensitivity check index: a simple, accurate method for assessing insulin sensitivity in humans. *Journal of Clinical Endocrinology and Metabolism*.

[18] Conti M, Morand PC, Levillain P, Lemonnier A (1991). Improved fluorometric determination of malonaldehyde. *Clinical Chemistry*.

[19] Catalán V, Gómez-Ambrosi J, Rotellar F (2007). Validation of endogenous control genes in human adipose tissue: relevance to obesity and obesity-associated type 2 diabetes mellitus. *Hormone and Metabolic Research*.

[20] Ceddia RB (2005). Direct metabolic regulation in skeletal muscle and fat tissue by leptin: implications for glucose and fatty acids homeostasis. *International Journal of Obesity*.

[21] Trostler N, Romsos DR, Bergen WG, Leveille GA (1979). Skeletal muscle accretion and turnover in lean and obese (*ob/ob*) mice. *Metabolism*.

[22] Matarese G (2000). Leptin and the immune system: how nutritional status influences the immune response. *European Cytokine Network*.

[23] Lord GM, Matarese G, Howard JK, Baker RJ, Bloom SR, Lechler RI (1998). Leptin modulates the T-cell immune response and reverses starvation—induced immunosuppression. *Nature*.

[24] Howard JK, Lord GM, Matarese G (1999). Leptin protects mice from starvation-induced lymphoid atrophy and increases thymic cellularity in *ob/ob* mice. *Journal of Clinical Investigation*.

[25] Fortuño A, San José G, Moreno MU, Díez J, Zalba G (2005). Oxidative stress and vascular remodelling. *Experimental Physiology*.

[26] Hunt JV, Smith CCT, Wolff SP (1990). Autoxidative glycosylation and possible involvement of peroxides and free radicals in LDL modification by glucose. *Diabetes*.

[27] Feillet-Coudray C, Rock E, Coudray C (1999). Lipid peroxidation and antioxidant status in experimental diabetes. *Clinica Chimica Acta*.

[28] Muzzin P, Eisensmith RC, Copeland KC, Woo SLC (1996). Correction of obesity and diabetes in genetically obese mice by leptin gene therapy. *Proceedings of the National Academy of Sciences of the United States of America*.

[29] Sivitz WI, Walsh SA, Morgan DA, Thomas MJ, Haynes WG (1997). Effects of leptin on insulin sensitivity in normal rats. *Endocrinology*.

[30] Chinookoswong N, Wang J-L, Shi Z-Q (1999). Leptin restores euglycemia and normalizes glucose turnover in insulin- deficient diabetes in the rat. *Diabetes*.

[31] Maingrette F, Renier G (2003). Leptin increases lipoprotein lipase secretion by macrophages: involvement of oxidative stress and protein kinase C. *Diabetes*.

[32] Bouloumié A, Marumo T, Lafontan M, Busse R (1999). Leptin induces oxidative stress in human endothelial cells. *FASEB Journal*.

[33] Beltowski J, Wójcicka G, Marciniak A, Jamroz A (2004). Oxidative stress, nitric oxide production, and renal sodium handling in leptin-induced hypertension. *Life Sciences*.

[34] Balasubramaniyan V, Nalini N (2007). Effect of leptin on peroxidation and antioxidant defense in ethanol-supplemented Mus musculus heart. *Fundamental and Clinical Pharmacology*.

[35] Sailaja JBK, Balasubramaniyan V, Nalini N (2004). Effect of exogenous leptin administration on high fat diet induced oxidative stress. *Pharmazie*.

[36] Gülen S, Dinçer S (2007). Effects of leptin on oxidative stress in healthy and Streptozotocin-induced diabetic rats. *Molecular and Cellular Biochemistry*.

[37] Hoeldtke RD, Bryner KD, McNeill DR, Warehime SS, Van Dyke K, Hobbs G (2003). Oxidative stress and insulin requirements in patients with recent-onset type I diabetes. *Journal of Clinical Endocrinology and Metabolism*.

[38] Harris ED (1992). Regulation of antioxidant enzymes. *FASEB Journal*.

[39] Watson AM, Poloyac SM, Howard G, Blouin RA (1999). Effect of leptin on cytochrome P-450, conjugation, and antioxidant enzymes in the *ob/ob* mouse. *Drug Metabolism and Disposition*.

[40] Ozata M, Uckaya G, Aydin A, Isimer A, Ozdemir IC (2000). Defective antioxidant defense system in patients with a human leptin gene mutation. *Hormone and Metabolic Research*.

[41] Pickup JC, Mattock MB (2003). Activation of the innate immune system as a predictor of cardiovascular mortality in Type 2 diabetes mellitus. *Diabetic Medicine*.

[42] Kjeldsen L, Cowland JB, Borregaard N (2000). Human neutrophil gelatinase-associated lipocalin and homologous proteins in rat and mouse. *Biochimica et Biophysica Acta*.

[43] Gabay C, Kushner I (1999). Acute-phase proteins and other systemic responses to inflammation. *The New England Journal of Medicine*.

[44] Catalán V, Gómez-Ambrosi J, Rodríguez A (2009). Increased adipose tissue expression of lipocalin-2 in obesity is related to inflammation and matrix metalloproteinase-2 and metalloproteinase-9 activities in humans. *Journal of Molecular Medicine*.

[45] Moylan JS, Reid MB (2007). Oxidative stress, chronic disease, and muscle wasting. *Muscle and Nerve*.

[46] Li Y-P, Chen Y, Li AS, Reid MB (2003). Hydrogen peroxide stimulates ubiquitin-conjugating activity and expression of genes for specific E2 and E3 proteins in skeletal muscle myotubes. *American Journal of Physiology*.

[47] Matthys P, Billiau A (1997). Cytokines and cachexia. *Nutrition*.

[48] Ebisui C, Tsujinaka T, Morimoto T (1995). Interleukin-6 induces proteolysis by activating intracellular proteases (cathepsins B and L, proteasome) in C_2_C_12_ myotubes. *Clinical Science*.

[49] Deval C, Mordier S, Obled C (2001). Identification of cathepsin L as a differentially expressed message associated with skeletal muscle wasting. *Biochemical Journal*.

[50] Li Y-P, Chen Y, John J (2005). TNF-*α* acts via p38 MAPK to stimulate expression of the ubiquitin ligase atrogin1/MAFbx in skeletal muscle. *FASEB Journal*.

[51] St-Amand J, Okamura K, Matsumoto K, Shimizu S, Sogawa Y (2001). Characterization of control and immobilized skeletal muscle: an overview from genetic engineering. *FASEB Journal*.

[52] Wittwer M, Flück M, Hoppeler H, Müller S, Desplanches D, Billeter R (2002). Prolonged unloading of rat soleus muscle causes distinct adaptations of the gene profile. *FASEB Journal*.

[53] Nath R, Kumar D, Li T, Singal PK (2000). Metallothioneins, oxidative stress and the cardiovascular system. *Toxicology*.

[54] Stevenson EJ, Giresi PG, Koncarevic A, Kandarian SC (2003). Global analysis of gene expression patterns during disuse atrophy in rat skeletal muscle. *Journal of Physiology*.

[55] Lecker SH, Jagoe RT, Gilbert A (2004). Multiple types of skeletal muscle atrophy involve a common program of changes in gene expression. *FASEB Journal*.

[56] Urso ML, Clarkson PM, Price TB (2006). Immobilization effects in young and older adults. *European Journal of Applied Physiology*.

[57] Morimoto RI (1993). Cells in stress: transcriptional activation of heat shock genes. *Science*.

[58] Lee C-K, Klopp RG, Weindruch R, Prolla TA (1999). Gene expression profile of aging and its retardation by caloric restriction. *Science*.

[59] Lawler JM, Song W, Kwak H-B (2006). Differential response of heat shock proteins to hindlimb unloading and reloading in the soleus. *Muscle and Nerve*.

[60] Selsby JT, Rother S, Tsuda S, Pracash O, Quindry J, Dodd SL (2007). Intermittent hyperthermia enhances skeletal muscle regrowth and attenuates oxidative damage following reloading. *Journal of Applied Physiology*.

[61] Liu Y, Gampert L, Nething K, Steinacker JM (2006). Response and function of skeletal muscle heat shock protein 70. *Frontiers in Bioscience*.

[62] Naito H, Powers SK, Demirel HA, Sugiura T, Dodd SL, Aoki J (2000). Heat stress attenuates skeletal muscle atrophy in hindlimb-unweighted rats. *Journal of Applied Physiology*.

[63] Selsby JT, Dodd SL (2005). Heat treatment reduces oxidative stress and protects muscle mass during immobilization. *American Journal of Physiology*.

[64] Senf SM, Dodd SL, McClung JM, Judge AR (2008). Hsp70 overexpression inhibits NF-*κ*B and Foxo3a transcriptional activities and prevents skeletal muscle atrophy. *FASEB Journal*.

[65] Garrido C, Gurbuxani S, Ravagnan L, Kroemer G (2001). Heat shock proteins: endogenous modulators of apoptotic cell death. *Biochemical and Biophysical Research Communications*.

[66] Kamradt MC, Chen F, Sam S, Cryns VL (2002). The small heat shock protein *α*B-crystallin negatively regulates apoptosis during myogenic differentiation by inhibiting caspase-3 activation. *The Journal of Biological Chemistry*.

[67] Nakamura T, Sakamoto K (2008). Forkhead transcription factor FOXO subfamily is essential for reactive oxygen species-induced apoptosis. *Molecular and Cellular Endocrinology*.

[68] Gomes-Marcondes MCC, Tisdale MJ (2002). Induction of protein catabolism and the ubiquitin-proteasome pathway by mild oxidative stress. *Cancer Letters*.

[69] McCarty MF (2006). Induction of heat shock proteins may combat insulin resistance. *Medical Hypotheses*.

[70] Aly KB, Pipkin JL, Hinson WG (1994). Chronic caloric restriction induces stress proteins in the hypothalamus of rats. *Mechanisms of Ageing and Development*.

[71] Heydari AR, You S, Takahashi R, Gutsmann A, Sarge KD, Richardson A (1996). Effect of caloric restriction on the expression of heat shock protein 70 and the activation of heat shock transcription factor. *Developmental Genetics*.

[72] Kurucz I, Morva A, Vaag A (2002). Decreased expression of heat shock protein 72 in skeletal muscle of patients with type 2 diabetes correlates with insulin resistance. *Diabetes*.

[73] Bruce CR, Carey AL, Hawley JA, Febbraio MA (2003). Intramuscular heat shock protein 72 and heme oxygenase-1 mRNA are reduced in patients with type 2 diabetes: evidence that insulin resistance is associated with a disturbed antioxidant defense mechanism. *Diabetes*.

[74] Figueiredo D, Gertler A, Cabello G, Decuypere E, Buyse J, Dridi S (2007). Leptin downregulates heat shock protein-70 (HSP-70) gene expression in chicken liver and hypothalamus. *Cell and Tissue Research*.

[75] Bonior J, Jaworek J, Konturek SJ, Pawlik WW (2006). Leptin is the modulator of HSP60 gene expression in AR42J cells. *Journal of Physiology and Pharmacology*.

